# Specificities of Myocardial Infarction and Heart Failure in Women

**DOI:** 10.3390/jcm13237319

**Published:** 2024-12-02

**Authors:** Milica Dekleva, Ana Djordjevic, Stefan Zivkovic, Jelena Suzic Lazic

**Affiliations:** 1 Faculty of Medicine, University of Belgrade, 11000 Belgrade, Serbia; dekleva.milica@gmail.com (M.D.); jsuzic@yahoo.com (J.S.L.); 2 Laboratory for Radiobiology and Molecular Genetics, VINČA Institute of Nuclear Sciences—National Institute of the Republic of Serbia, University of Belgrade, 11000 Belgrade, Serbia; 3 Clinic for Cardiology, Institute for Cardiovascular Disease “Dedinje”, 11000 Belgrade, Serbia; zivkovic.stefan@gmail.com; 4 Clinic for Internal Medicine, Cardiology Department, University Clinical Hospital Center “Dr. Dragisa Misovic-Dedinje”, 11000 Belgrade, Serbia

**Keywords:** myocardial infarction, sex differences, left ventricular remodeling, heart failure

## Abstract

Substantial evidence from previous clinical studies, randomized trials, and patient registries confirms the existence of significant differences in cardiac morphology, pathophysiology, prevalence of specific coronary artery disease (CAD), and clinical course of myocardial infarction (MI) between men and women. The aim of this review is to investigate the impact of sex or gender on the development and clinical course of MI, the mechanisms and features of left ventricular (LV) remodeling, and heart failure (HF). The main sex-related difference in post-MI LV remodeling is adverse LV dilatation in males versus concentric LV remodeling or concentric LV hypertrophy in females. In addition, women have a higher incidence of microvascular dysfunction, which manifests as impaired coronary flow reserve, distal embolism, and a higher prevalence of the no-reflow phenomenon. Consequently, impaired myocardial perfusion after MI is more common in women than in men. Regardless of age or other comorbidities, the incidence of reinfarction, hospitalization for HF, and mortality is significantly higher in females. There is therefore a “sex paradox”: despite the lower prevalence of obstructive CAD and HF with reduced ejection fraction (HFrEF), women have a higher mortality rate after MI. Different characteristics of the coronary network, such as plaque formation, microvascular dysfunction, and endothelial inflammation, as well as the prolonged time to optimal coronary flow restoration, secondary mitral regurgitation, and pulmonary vascular dysfunction, lead to a worse outcome in females. A better understanding of the mechanisms responsible for MI occurrence, LV remodeling, and HF in men and women would contribute to optimized patient therapy that would benefit both sexes.

## 1. Introduction

In addition to the specific characteristics of patients with myocardial infarction (MI), there are a number of indications of gender differences in the etiology, clinical presentation, and outcome of MI. The aim of this review is to examine the impact of gender on the development and clinical course of myocardial infarction, the mechanisms and characteristics of left ventricular (LV) remodeling, and heart failure (HF). It is important to clarify that “sex” and “gender” are different terms. “Sex” refers to the biological differences between men and women and includes factors such as chromosomes, hormones, and reproductive function. In contrast, “gender” encompasses an individual’s lifestyle, including social, cultural, and institutional context; physical activity, level of education, mental health and self-perception. It should be noted that the boundary between sex and gender is not absolute; both sex and gender are interrelated and subject to change. It is well established that biological characteristics can be influenced by gender and that sexual identity can be influenced by life circumstances [1,2].

It is already known from previous studies, randomized clinical trials, and patient registries that there are significant sex-based differences in patients with MI [3,4,5]. Women with coronary artery disease (CAD) are older, have more comorbidities and risk factors, but have less advanced epicardial CAD, a lower number of previous MIs, percutaneous interventions, or re-vascularization. However, despite these data, they have a higher incidence of HF and a higher mortality rate [5].

## 2. Sex Differences in General Risk Factors

Ageing: Several risk factors contribute to the differences between men and women with CAD. Women develop MI and heart failure with preserved ejection fraction (HFpEF) 7–10 years later than men [6]. Myocardial infarction is 3–4 times more common in men, but after the age of 75, the female gender predominates [7]. Recently, however, studies have shown that the annual incidence of acute MI hospitalizations (from 1995 to 2014) has increased significantly in young women (*p* for trend = 0.002), but has decreased in young men (35–54 years) [8,9].

Cardiometabolic: Although there is a trend towards an increase in acute MI in young women, the differences between men and women are significantly influenced by sex hormones. Younger-aged women with acute MI have multiple risk factors and comorbidities. Cardio-metabolic risk factors such as hypertension, obesity, and tobacco smoking have a greater impact on the occurrence of the disease in women than in men. According to the ICACS-TC registry, the prevalence of these factors has a significant impact on higher early mortality [10,11]. Diabetes mellitus (DM) is more likely to contribute to mortality in women than in men with CAD [8,9,10,11]. Women with DM are more likely to have maladaptive LV remodeling with increased LV thickness and LV mass index after myocardial infarction [12]. Secondary prevention of common risk factors is less effective in women than in men [13].

## 3. Female Specific Risk Factors

In addition to the commonly known, there are other significant and proven risk factors for LV remodeling and HF in the female population, such as: Preterm delivery, hypertensive disorders of pregnancy, gestational DM, breast cancer treatments such as radiation or chemotherapy, and autoimmune diseases such as rheumatoid arthritis or systemic lupus [13].

Pregnancy-related disorders are associated with the risk of cardiovascular disease (CVD). The occurrence of hypertensive and metabolic pregnancy disorders correlates with the occurrence and severity of CVD later in life [14].

It is already known that systemic diseases are more common in women. New data show that chronic inflammation and microvascular injury lead to and accelerate CAD [15].

There is an increased risk of CAD in women undergoing radiotherapy or chemotherapy for breast cancer [16]. Incidental or therapeutic exposure of the heart to ionizing radiation is associated with accelerated coronary atherosclerosis and subclinical or clinical LV dysfunction. Breast cancer patients treated with chemotherapy are at risk for two types of cardiotoxicity (anthracycline-like and trastuzumab-like agents), both of which are dose-dependent. Delayed cardiotoxicity can range from LV dysfunction to overt HF, arrhythmias, or ischemia [16].

Risk factors such as obesity and physical inactivity have a greater impact on the development and clinical course of CAD, LV remodeling, and HF in women than in men [12,17].

Women are more frequently affected by depression, which contributes significantly to the development of MI. Depression is also an important factor in the patient’s outcome and prognosis. Depression represents a therapeutic challenge in post-MI patients, as the use of antidepressants in patients with CAD could pose a therapeutic dilemma [18]. The results of several studies show that the use of tricyclic antidepressants in patients with depression is associated with an increased risk of CAD [19]. In these patients, selective serotonin reuptake inhibitors (SSRIs) proved to be the treatment of choice. Pizzi et al. conducted a meta-analysis of 6 randomized trials and 7 reports examining the effect of SSRI medications in patients with CAD and depression. They concluded that SSRI treatment may be beneficial in patients with CAD [18].

**Figure 1:**
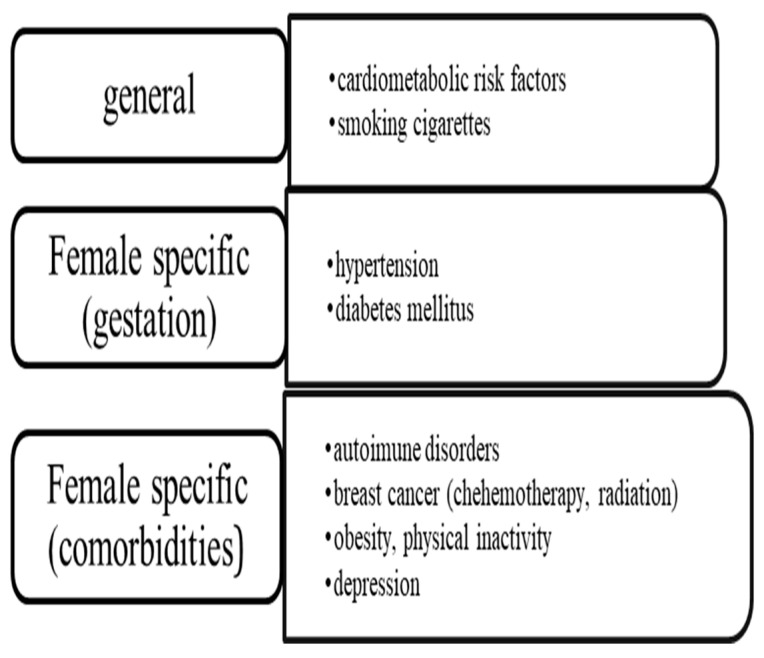
Risk factors for myocardial infarction.

In summary, all of the above risk factors, summarized in Figure 1, have been associated with a higher risk of morbidity and mortality in women with CAD [14,15,16,17].

## 4. Reperfusion in Females

It is known that there are differences between the sexes in the mechanisms of occurrence and response to ischemic injury, such as ischemic preconditioning and platelet aggregation [20,21]. However, in women, there is prolonged reperfusion with suboptimal coronary flow recovery, differences in plaque characteristics (diffuse and non-obstructive) and impaired pulmonary vascular function, chronic inflammation, and more frequent secondary mitral regurgitation, which contribute to a poorer prognosis in women [7]. In a prospective, observational cohort study of 1465 young patients aged 18 to 54 years, women had more frequent reperfusion delays than men of the same age [9,22]. The higher endothelial shear stress in women can be explained by a smaller diameter of the epicardial coronary arteries and thus a higher rate of resting blood flow [7,23]. The WISE study found changes in the microcirculatory network and diffuse coronary artery atherosclerosis, which may explain a higher number of angina pectoris episodes and revascularization procedures after MI in women [24,25].

The term “gender paradox” was described in the VIRGO study, showing that a promising response to reperfusion in young women is not accompanied by better clinical outcomes [22]. Despite similar infarct size and LV function, women have higher in-hospital complications, major bleeding, and 1-year mortality from HF, as well as higher rates of HF and recurrent MI development [4,21,22,23,24,25,26]. In patients with ST-elevation myocardial infarction (STEMI), sex is the predictor of the higher mortality, independent of age and other risk factors [5]. Although women have better angiographic status, i.e., a lower incidence of critical stenosis and better coronary flow rates before primary percutaneous coronary intervention (pPCI), younger women (<60 years) with STEMI have a two times higher mortality rate than men [22,23]. In women, the infarction zone is more often smaller, as well as the degree of fibrosis and the size of the scar, and therefore the degree of thinning of the myocardial wall and dilation of the left ventricle [21]. After effective reperfusion, the myocardium in the infarction zone may increase its contractile function and recover regional and global LV function. But limited coronary reserve and the microvascular dysfunction in females are associated with cardiomyocyte damage leading to diastolic LV dysfunction and the HFpEF phenotype [6,27].

Therefore, the optimal reperfusion and revascularization must include not only early and sustained epicardial patency but also optimal microvascular flow and tissue reperfusion.

## 5. Non-Obstructive vs. Obstructive Coronary Artery Disease: MINOCA vs. MIOCA

The difference between the sexes in patients with MI lies in the fact that obstructive CAD (MIOCA) is a significantly more frequent cause of MI in men, while it is a non-obstructive disease that is more frequent in women (MINOCA). Despite this finding, women have more complications during in-hospital stays and higher mortality after 4.62 years of follow up [27]. Diagnosis of MINOCA, a condition that occurs in 6–10% of all MI [28], requires that in addition to all the accepted criteria for acute infarction, there is evidence on coronary angiogram of non-critical stenosis (<50%) of the epicardial coronary arteries [28]. Patients with MINOCA tend to be younger and less likely to have general risk factors other than hypertension [29]. It is important to separate patients with normal coronary arteries or minimal luminal irregularities (≤30% stenosis) from those with mild to moderate coronary atherosclerosis (30–50%). In such cases, fractional flow reserve testing (FFR) can be a valuable diagnostic tool. The mechanisms underlying the development of MINOCA are numerous and complex. Today, it is considered that the most important are: processes in the epicardial coronary vessel (rupture/fissure of small plaque, spontaneous coronary artery dissection, epicardial vasospasm, and in situ thrombosis), coronary microvascular disease, and the increased oxygen supply and/or oxygen demand, usually in LV hypertrophy [28,30]. Plaque disruption is a common mechanism in MINOCA patients, and it includes plaque rupture, plaque erosion, and calcific nodules. The use of optical coherence tomography or intravascular ultrasound imaging can reveal the etiology of MINOCA and trace the therapeutic approach [28,30]. Hypercoagulable state is not a mandatory condition for coronary thrombosis or embolism occurrence in MINOCA state [30]. Coronary vasospasm is another common cause of MINOCA; this and several types of coronary microvascular dysfunction, both endothelium-dependent and independent, can be detected by invasive and non-invasive testing [28,29,30,31,32,33]. The international guidelines currently state that there are sex-specific differences in the clinical presentation of male and female patients with AMI [34]. Women are more likely to have atypical symptoms such as epigastric pain, dyspepsia, fatigue, neck pain, or shortness of breath, and in 43% of cases the myocardial infarction is asymptomatic. In comparison, asymptomatic MI in men is present in 24% of cases [35]. Accurate recognition and interpretation of clinical symptoms has major implications for the diagnosis, treatment, and management of patients with MI. However, the sub study conducted by Ferry et al. demonstrated that typical symptoms are more common and have a higher predictive value in women than in men with MI. In fact, the authors suggested that assessing the female patients using a cluster of symptoms, including pain nature, location, radiation, and presence of other symptoms, may be more clinically relevant than focusing on a single symptom [34]. Given that female patients are less affected by CAD, the expected outcomes should be favorable, but they are often underdiagnosed or have diagnostic and treatment delays and receive less evidence-based treatment [35]. The recently published study has shown that biological sex differences, such as sex hormones, genetic and neurological factors, cardiac innervation, and pain sensitivity, on the one hand, and gender differences in psychological status, especially depression, anxiety, post-traumatic stress, and socioeconomic circumstances, on the other, are the main factors for the different clinical features of MI [35]. The underrepresentation of women in large randomized trials means that there are no guidelines that take sex differences into account, leading to a gender bias among clinicians, healthcare professionals, and women themselves when it comes to treating suspected CAD, especially at a younger age with a poor prognosis [35].

Clinical presentation of MINOCA is dominantly MI with non-ST-elevation myocardial infarction (NSTEMI) and is present in approximately two-thirds of cases. MINOCA patients have similar mortality with MIOCA patients, but women with MIOCA have a higher mortality rate within one-year post-discharge from the hospital than men [27,30]. In symptomatic female patients with MINOCA, the most common cardiac event is HFpEF, with an approximately 10-fold higher incidence than in asymptomatic women with MIOCA [30,36].

A recent study evaluated the predictive value of gender in the prognosis of MINOCA and the difference in survival and major adverse cardiac events (MACE) development during five years of follow up between MINOCA and MIOCA groups [28]. Canton et al. showed that the incidence of MACE in women was significantly higher in both groups (MINOCA and MIOCA). In younger female patients aged <70 years in the MINOCA group, hospitalization for HFpEF and recurrent MI are the most common. In a subgroup of MINOCA patients aged <70 years, female gender was an independent predictor of MACE [28]. Β-blockers and statins in secondary prevention have been shown to have a beneficial therapeutic effect in women and to improve survival after MINOCA [29,32]. Unfortunately, these drugs are not regularly prescribed to women after MINOCA when they are discharged from the hospital [29]. All the aforementioned differences between male and female patients with MI are shown and summarized in Table 1.

## 6. Sex-Related Mechanisms of Left Ventricular Remodeling

### 6.1. The Cellular and Extracellular Changes in the Early and Late Phases of Myocardial Infarction

LV remodeling after MI involves the morphological, functional, and bio-humoral changes that occur in myocytes and extracellular space in the infarcted and peri-infarcted zones [7,12]. Immediately after the ischemic injury in acute MI, myocardium changes its structural and mechanical properties and begins the processes of the deposition of collagen, the excitation–contraction uncoupling, apoptosis, and fibrosis in order to preserve the heart function, reduce the infarct zone, and minimize the myocardial stress. The healing process in which inflammation and fibrosis are combined at an early stage of remodeling plays a compensatory role to alleviate regional dysfunction and establish normal global LV function.

It is already known, from previous studies, that the characteristics of normal myocardium are different between men and women [37]. Furthermore, post-mortem data studies suggested that males have a 10-fold higher rate of apoptosis than females [36,37]. Furthermore, other additional processes of the infarct healing and remodeling, such as tissue repair, degradation of extracellular matrix (ECM), and myocardial slippage, are different in men and women [38,39]. Gene mutations responsible for different remodeling phases and processes are expressed at different levels among males and females [40]. A very important role in the described gender differences during the post-MI remodeling process is played by the circulating sex female hormone estrogen [41]. Deficiency of estrogen is associated with high vascular stiffness and therefore hypertension, diastolic LV dysfunction, and HFpEF development [41,42,43]. Estrogen also modulates natriuretic peptides and accelerates angiogenesis, which stimulates oxygen demand in the hypertrophic heart [39,41,43].

Delayed apoptosis in females, deposition of fibrosis and collagen, and higher levels of inflammation may contribute to LV dysfunction and late post-infarction complications in females [37,38,39,40,44].

### 6.2. Hemodynamic and Functional Patterns of Left Ventricular Remodeling After Myocardial Infarction

The course of the remodeling process depends on the degree of peri-infarction apoptosis and necrosis [38,43]. It has been proposed that the expansion of the infarction zone lasts hours after MI, but extension that is due to changes in non-ischemic myocardium is ongoing during weeks and months after MI [43]. All structural and mechanical changes in myocardium lead to different volume pressure relationships and generate a dynamic pattern of remodeling after MI. The initial adaptive phase enables the heart to normalize wall stress and preserve cardiac output, while in the chronic course hypertrophy and dilatation occur with volume and/or pressure overload. Different LV geometric patterns are associated with distinctive pathophysiologic modalities, which are very important for risk stratification in patients after MI [12,39,41,43,45,46].

In women, predominantly, the process of LV hypertrophy after MI is most likely associated with metabolic and functional changes, flow disorders, and the development of HF [12,46,47,48].

In a VALIANT echocardiographic sub-study, authors showed that concentric LV hypertrophy (increased basal LV mass and index of relative wall thickness) carries the greatest risk of advanced cardiovascular events after MI, including death [49,50]. Hemodynamic characteristics of LV remodeling in women are better regulated and tolerated with a volume-pressure ratio, lower fibrosis and myocardial dilation, but elevated wall stress and LV filling pressure [43,48]. The detection of LV hypertrophy in women after MI has much greater significance for the outcome and stratification of risk than in men [48,49,50]. Progression toward HFpEF occurs in women more often than in men through the faster intermediate step between condition and disease [48,49,50,51].

### 6.3. Sex Difference in Reverse Left Ventricular Remodeling

The term “myocardial recovery” has been recently introduced and denotes that long-term treatment with neurohumoral blockage (sympathetic, renin angiotensin aldosterone, and inflammatory cytokine system) can alleviate the process of maladaptive remodeling and lead to the return of myocardial structure and function [45,52]. Thus, the changes in LV volume occur secondary to the myocardial recovery process, i.e., reverse remodeling (RR) is a complex process of restoration of chamber geometry and function, including corrections of molecular and transcriptional abnormalities. Essential changes in RR include a decrease in the size of myocardial cells and collagen amounts, an intense microvascular network, hemodynamic optimization, and restoration of cardiac biomarkers and exercise capacity [52].

The mechanisms of RR are not entirely known, but the most responsible processes are thought to be in the ECM, i.e., in the altered form of collagen. These reverse changes occur due to the beneficial effects of exercise, ACE inhibitors, and β-blocker therapy [45]. In patients with HF with reduced ejection fraction (HFrEF), RR was projected and confirmed [52]. It has been observed that RR leads to a better clinical outcome and may occur spontaneously or with myocardial revascularization, surgical, pharmacological, or device therapy [53]. The previously generally accepted term “ventricular remodeling”, which included LV dilatation with altered topography and function after coronary artery occlusion, should be aligned with the terms HFrEF and HFpEF in order to indicate patterns of remodeling, hemodynamic and gender differences, and determine the correct therapeutic approach [52,53].

## 7. Risk Factors in Heart Failure Development

The risk of HF development after MI is higher in women, who have a worse outcome and survival compared to men [20]. Sex disparities are present among patients with HF with ischemic or non-ischemic origin across various aspects, including epidemiology, risk factors, pathophysiological mechanisms, diagnostic approach, clinical courses, comorbidities, treatment strategies, and risk stratification [54]. The prevalence of obstructive CAD in men is the main reason for the maladaptive LV remodeling and HFrEF development [12]. Women have a higher percentage of preserved systolic function, i.e., HFpEF, while in men the systolic function is reduced (HFrEF). Indeed, half of the women and only one-third of men with HFpEF are presented with signs and symptoms of HF [51,54]. Some of the general risk factors, such as DM, obesity, hypertension, dyslipidemia, and smoking, appear to be more important in women than in men for the development of the specific phenotype of HF [55]. Although the prevalence of hypertension and smoking in women is lower compared to men, both are associated with a higher risk of HF development [54]. Some of the risk factors specific to women, such as hypertensive disorder of pregnancy (HDP), eclampsia, or pre-eclampsia, have been shown to transiently change cardiac structure and function and are associated with a higher severity of HF in later life [54,55]. Anemia with iron deficiency, which occurs more often in women, in HF conditions, favors the development of cardiorenal syndrome and significantly worsens the prognosis [55]. Recent studies showed that nulliparity and shorter total reproductive duration are associated with a higher risk of HF occurrence [56]. The causal relationship and the role of estrogen deficiency have not been sufficiently investigated.

## 8. Types of Heart Failure: Gender Differences

Recent research has confirmed that LV diastolic dysfunction is more pronounced in women, but, at the same time, with a smaller or larger limitation of systolic function [42,44]. Several factors contribute to this associated dysfunction: systemic and pulmonary vascular function, right ventricular (RV) function, autonomic tone, and chronotropic reserve [42,43]. Microvascular dysfunction plays a key role in the remodeling process and HFpEF development [36,42,44]. Microvascular injury (functional and structural), pro-inflammatory conditions, and endothelial dysfunction contribute to the change of cardiomyocytes and the increase in fibrous tissue, resulting in diastolic dysfunction [28,36]. Clinical presentation in women is more often a stiffer heart with a smaller stroke volume, consequent limitation of diastolic LV reserve with higher wedge and LV filling pressure, and impaired ventricular–vascular coupling [39,42,43]. Duca et al. demonstrated that men with HFpEF have higher LV end-diastolic volume (LVEDV) and stroke volume but also more often right ventricular (RV) dilatation and impaired RV function and lower aerobic capacity compared to women [42]. In the meta-analysis of 10 randomized studies of patients with acute STEMI treated with pPCI, Kosmidou et al. showed that women had significantly higher LV ejection fraction (LVEF) measured by NMR technique, but there was no difference in infarction size, measured by SPECT, among men and women [7]. In comparison to men, women have fewer comorbidities (atrial fibrillation (AF): 68% vs. 55%; anemia: 73% vs. 61%; sleep apnea: 20% vs. 5%; chronic obstructive pulmonary disease (COPD): 47% vs. 27%, respectively) [6,12,42,44]. Recent data showed that previous MI in patients with HFpEF was associated with greater cardiovascular and sudden death risk and worse outcomes. Compared to men, women had a significantly higher risk of all causes of death and HF hospitalization after a 12-month period [6].

Heart failure from other different origins showed sex related trends in development, diagnosis, and clinical expression. The incidence of hypertrophic cardiomyopathy (HCM) is different between sexes: women account for 35–40% of HCM patients. Diagnosis of HCM in women is usually made at an older age with more severe symptoms and a higher risk of HF, AF, and stroke progression, but the incidence of sudden cardiac death is similar in both men and women. A higher percentage of women carry sarcomere gene variants responsible for clinical presentation and progression [54,57]. Approximately, Fabry disease (FD) is diagnosed in 0.9% of patients with HCM, in whom hypertrophy and fibrosis progress rapidly, leading to HF predominantly in men [58]. Cardiac amyloidosis, including wild-type transthyretin and light chain type presents more often in men (80–90%), potentially due to sex differences in myocardial fibril composition. Sex hormones are active in this condition with a negative influence of 5α-dihydrotestosterone compared to estrogen in an animal model [59]. Dilatation of LV with systolic dysfunction without evidence of CAD or any other known disease is called idiopathic dilatative cardiomyopathy (DCM). Genetic testing for identifying DCM showed no sex variations, but serious complications and worse outcomes occur more often in men [60]. It is also evident that arrhythmogenic cardiomyopathy occurs predominantly in men. Sex differences and, at the same time, the predisposing factors for a poor outcome in arrhythmogenic cardiomyopathy can be a high level of testosterone and greater physical efforts [59]. Systemic autoimmune disorders such as rheumatoid arthritis (RA) and systemic lupus erythematosus (SLE) are more prevalent in women. The main causes of HF development in these diseases are chronic inflammatory processes and impaired microcirculation that cause tissue destruction [61]. Peripartum cardiomyopathy (PPCM) clinically presents with symptoms and signs of HF at the end of pregnancy or several months after delivery. The etiology of PPCM is heterogeneous with evidence of several risk factors, such as multiple pregnancies, family history, ethnicity, DM, hypertension, viral myocarditis, and autoimmune disease [56,62]. Heart failure is a serious clinical outcome of myocarditis and progressive dilated cardiomyopathy (DCM), in which inflammation is the main pathogenic mechanism. Studies show that myocarditis and DCM occur more frequently in men (women to men 1:3). In women, dyspnea is the predominant symptom, and unlike men, they are over 50 years of age and have a better regulatory immune response and lower levels of biomarkers, remodeling, and fibrosis, all of which lead to DCM and HF, compared to men [63]. The therapeutic approach for myocarditis and DCM follows HF guidelines, but standard medications for HF show differences in efficacy between men and women [63,64].

Advanced heart failure refers to patients with severe heart failure (NYHA Functional Class III-IV) despite guideline-directed drug therapy, in which case mechanical supportive therapy is indicated. There is ample evidence that there are many sex differences in device implantation and heart transplant therapy [65]. The literature reports: fewer appropriate ICD shocks in women compared to men, greater benefit of CRT, similar survival benefit of left ventricular assist device (VAD) but higher risk of neurologic adverse events, poorer survival awaiting heart transplantation in patients with similar medical urgency but slightly better survival than men after transplantation [65,66]. Possible reasons for this include the smaller diameter of the heart and vessels, a greater tendency to bleed during invasive procedures, and a more severe form of heart failure at the time of device implantation, as well as mismatching of donor and recipient [65,66,67]. Sex differences in patients with heart failure are presented in Table 2.

## 9. Variants in Cardiovascular Pharmacotherapy

It is well known that there are sex differences in the efficacy and safety of cardiovascular drugs, but in everyday practice these findings are not taken into account.

Biological differences among sexes in body composition, fluctuations in sex hormones, clinical characteristics, and specificity of MI and remodeling pattern significantly influence the effective response of cardiovascular therapy [62,64]. It is becoming increasingly clear that the pharmacokinetics, pharmacodynamics, and pharmacogenetics of several drugs differ between the sexes. These include cardiovascular drugs, the cornerstones of pharmacologic therapy prescribed after myocardial infarction to preserve cardiac function and infarct-related arterial patency, such as beta-blockers, renin–angiotensin–aldosterone inhibitors, and antithrombotic drugs. Some physiological differences between men and women may influence drug metabolism. The distribution of the hydrophilic and lipophilic compartments is different; women have a higher proportion of body fat and a lower plasma volume, and the elimination times are longer with increased blood concentrations of the drugs. Therefore, the frequency of adverse drug reactions is higher in women [68]. Higher gastric pH and lower intestinal fluid volume lead to lower oral bioavailability and reduced or delayed absorption of beta-blockers (metoprolol), calcium channel blockers (verapamil), and enteric-coated aspirin. In women, lower glomerular filtration and tubular secretion were found, resulting in slower renal clearance of propranolol, metoprolol, and verapamil [69]. There are differences between men and women in hepatic metabolism and in the activity of membrane transporters in the intestine. Therefore, several cardiovascular drugs, such as labetalol, propranolol, verapamil, the ARB inhibitor losartan, and the platelet aggregation inhibitor ticagrelor, achieve improved bioavailability and absorption [68] (Table 3). Female patients are more susceptible to electrolyte imbalance after taking therapy as well as to the pro-arrhythmogenic action of some antiarrhythmics with QT interval prolongation and the appearance of malignant arrhythmia, which may all be the reason for the worse outcome and prognosis in women after MI [69].

Despite the important role of sex hormones in women and promising experimental studies, some clinical trials have shown that estrogen replacement therapy was associated with a higher incidence of CAD and thromboembolism without a significant impact on cardiovascular mortality in women with a history of MI [68].

Significant biological differences, such as smaller vessel size and higher prevalence of MINOCA in women, may limit the therapeutic benefit of PCI and standard drug therapy for MI. The ILUMIEN IV trial confirmed the clinical benefit and predictive power of intravascular imaging in PCI and the positive impact of OCT guidance [70]. It is known that the risk of cerebral hemorrhage after PCI is higher in women, and anticoagulation with unfractionated heparin also increases the risk of bleeding [68]. New pharmacological strategies to limit ischemia-reperfusion injury are the subject of numerous past and ongoing experimental studies and clinical trials. Since superoxide dismutase activity is higher in the heart of women than in men, new drugs targeting oxidative stress are the subject of ongoing pilot studies [68,69]. The different stimulation of the A1 receptor and the different response of the endothelium in men and women indicate that the cardioprotective effect of adenosine is lower in women [63]. Drugs that target inflammation, such as doxycycline, rituximab (monoclonal antibody), and those that act on thrombosis (zalunfiban) (GPIIb/IIIa inhibitor), are the subject of research, as it is known that the platelets and fibrinolytic system are more reactive in women than in men [68]. The new drugs that have found clinical application are those that act on cardiometabolic factors, i.e., lipid-lowering drugs such as evolocumab (monoclonal antibody against proprotein convertase subtilisin/kexin type 9-PCSK9), which is less effective in lowering LDL cholesterol in women than in men, and inhibitors of sodium-glucose co-transporter 2 (SGLT2) (dapagliflozin and empagliflozin), which also showed less clinical benefit in women with HF than in men [69,71].

EMA601 is a novel antiplatelet drug with the ability to inhibit glucoprotein VI (GPVI), a platelet collagen/fibrin receptor, and thus prevent or treat arterial thrombosis and thrombo-inflammatory processes in high-risk patients. Experimental studies and ex vivo results are likely to be supported by clinical trials such as the LIBERATE study to allow this high potential GPVI inhibitor to be used clinically [72]. A step towards personalized therapy is undoubtedly the use of colchicine in cardiology practice, slowing the process of atherosclerosis in patients with TET2 gene mutation who exhibit clonal hematopoiesis [73]. An important contribution to understanding the mechanisms of sex-based differences in cardiovascular disease are the results and conclusions of the study by Titova et al. who investigated how circulating cardiometabolic proteins affect the risk of myocardial infarction [74]. The authors concluded that forty-five proteins were associated with the occurrence of incident MI, and 13 of the protein associations were sex specific, with the majority affecting women. The proteins identified in this study and the observed sex-specific differences in the associations between proteins and future MI with potential explanations shed light on the development of MI and could form the basis for the development of personalized medicine and meet the unique needs of women with MI [74,75].

Infarct-related cardiogenic shock (CS) is one of the most severe complications with very limited therapeutic options and a mortality rate of up to 50% within the first 30 days [76]. In a recent study by Wang et al., the authors postulated that sex specific performances of the ORBI score might differ and that adjusting for these differences could improve its predictive power. They also showed that the novel SEX-SHOCK score, in which several calculation components differ between men and women, provides better prediction of intrahospital CS in women and men across the spectrum of acute coronary syndrome. The main variables selected to calculate the score in women compared to men were CRP, ST-segment elevation, LVEF, and creatinine level [77].

Recent studies suggested that women with HFrEF might need lower doses of ACE inhibitors or angiotensin receptor blockers (ARBs) and β-blockers than men and do not experience any additional benefits with titration to the peak dose of these medications [78,79]. The therapeutic benefit of sacubitril-valsartan (ARNI drug) in reducing the risk of HF hospitalization is more significant in women than in men. The same study showed that valsartan monotherapy (without a neprilysin inhibitor) of HFpEF resulted in lower efficacy in women compared to men [44]. It has also been shown that the response to HF resynchronization therapy is significantly better in women [67].

The therapeutic efficacy of digitalis is significantly lower in women, and it was even suggested it may even have a detrimental effect in women with HFrEF and increase the risk of death from any cause [80]. However, beneficial effects of digitalis might be very important in the therapeutic approach of HFrEF in men. In patients with HF included in the TOPCAT study, it was shown that the recommended medications were equally effective in all types of HF regardless of LVEF in women, while in men this was only the case with HFrEF [81].

In everyday practice, a smaller number of women than men receive timely and adequate therapy in primary and secondary prevention of atherosclerotic disease of the coronary arteries. Women with similar cardiovascular risk receive less often antiplatelet and lipid-lowering drugs than men, but also neurohormonal antagonists (blockers of the renin–angiotensin system, β-blockers, and mineralocorticoid receptor antagonists) [64,79]. Women are less likely than men to be treated with coronary reperfusion therapy in the form of a PCI procedure or fibrinolysis. Women are less likely than men to participate in and complete cardiac rehabilitation programs (Table 3).

Some of the negative reactions to drugs are more widespread and severe in women. Personalized pharmacological treatment with dose-adjustments in the female population would address the need for true optimal therapy for men and women.

## 10. Conclusions

Gender differences in patients with myocardial infarction are related to the risk factors that affect the occurrence of MI, mechanisms of myocardial damage, characteristics of coronary lesions and microcirculation, left ventricular remodeling, and development of heart failure. The complex interaction of cellular, extracellular, neurohormonal, inflammatory, and genetic factors in acute infarction is likely the basis for the diversity of occurrence, clinical presentation, hemodynamic response, and outcomes between men and women. The assessment of these integrative factors, together with cardiovascular therapy, provides a better insight into all processes of LV remodeling and HF development.

## Figures and Tables

**Table 1:** Sex and gender differences in patients with myocardial infarction.

Characteristics	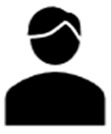	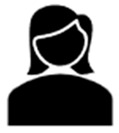
Risk Factors	Hypercholesterolemia, hypertension, diabetes mellitus, smoking	+Autoimmune diseases (RA, SLE) hormonal imbalance, chronic inflammation, obesity, comorbidities, physical inactivity, PPCM
Clinical presentation	Typical chest pain, breathlessness	Atypical symptoms (epigastric pain, nausea, fatique, neckpain), asymptomatic
Type of MI	MIOCA, dominant STEMI	MIOCA and MINOCA, dominant NSTEMI
Coronary circulation	Epicardial coronary arteries, larger diameter, critical stenosis	Smaller diameter, endothelial stress, SCAD, coronary vasospasm, impaired microcirculation
Reperfusion	Fast, optimal, less ischemic reperfusion injury	Suboptimal, late, more “no reflow” phenomenon
Infarction zone	Large infarction zone expansion, wall thinning, LV dilatation	Smaller scar, non-ischemic zone extension, less fibrosis
Plaque	Eccentric, obstructive	Diffuse, non-obstructive
Treatment	Primary PCI procedure, Standard therapy	Less evidence-based therapy, treatment delays, negative reactions to the drugs

MI—myocardial infarction, RA—rheumatoid arthritis, SLE—systemic lupus, PPCM—postpartum cardiomyopathy, MIOCA—myocardial infarction with obstructive coronary arteries, MINOCA—myocardial infarction with non-obstructive coronary arteries, STEMI—ST elevation myocardial infarction, NSTEMI—non-ST elevation myocardial infarction, LV—left ventricular, SCAD—spontaneous coronary artery dissection, PCI—percutaneous coronary intervention.

**Table 2:** Sex differences in patients with heart failure.

Characteristics	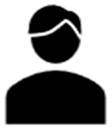	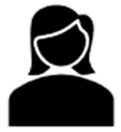
Risk Factors	CAD, hypertension, diabetes mellitus, smoking	+Hypertensive disorder in pregnancy Eclampsia, anemia with iron deficiency, rheumatic diseases
Type of HF	HFrEF: DCM, myocarditis, HCM, Fabry disease, AL	HFpEF, HFmEF: HF in RA, SLE, PPCM
LV remodeling and function	LV dilatation, eccentric remodeling Systolic Dysfunction, lower EF	Concentric LV remodeling, Dominant Diastolic LV dysfunction, higher EF
Hemodynamic	Higher preload, RV dilatation and dysfunction	Smaller SV, high wedge and LV filling pressure, impaired LV-vascular coupling
Mechanisms	Obstructive CAD, maladaptive LV remodeling	Microvascular injury, pro-inflammatory condition, endothelial dysfunction

HF—Heart Failure, DCM—dilatative cardiomyopathy, CAD—coronary artery disease, EF—Ejection Fraction, RV—right ventricular, HFrEF—heart failure with reduced ejection fraction, HCM—hypertrophic cardiomyopathy, AL–amyloidosis, HFpEF—heart failure with preserved ejection fraction, HFmEF—heart failure with mid-range ejection fraction, SV—stroke volume.

**Table 3:** Major female characteristics of the therapeutic approach.

Physiological Characteristics	Pharmacodynamics	Standard Therapy	Drug Response
Higher lipophilic compartment Lower plasma volume Higher gastric pH Smaller volume of small intestinal fluid and lower P-gp activity Lower glomerulal filtration rate	Increased blood concentration Prolonged times elimination Lower oral bioavaiability Reduced and delayed drugs absorption	Treatment delays (pPCI) Low rate of receiving reperfusion Low prescription rate of BB, antiplatelet drugs, ACE inhibitors and statin Incomplete cardiac rehabilitation	More adverse drug reactions Higher risk of severe bleeding Greater reduction in HR and BP during BB therapy Hypo Na, HypoK as a result of diuretics Lower dose of ACE inhibitors, ARNI and BB, better effect of CRT

ACE—angiotensin-converting enzyme, BB—β-blockers, pPCI—primary percutaneous intervention, HR—heart rate, CRT—cardiac resynchronization therapy, ARNI—Angiotensin receptor-neprilysin inhibitors, BP—blood pressure.

## References

[1] Reale C., Invernizzi F., Panteghini C., Garavaglia B. (2023). Genetics, sex, and gender. J. Neurosci. Res..

[2] Short S.E., Yang Y.C., Jenkins T.M. (2013). Sex, gender, genetics, and health. Am. J. Public Health.

[3] Vaccarino V., Parsons L., Peterson E.D., Rogers W.J., Kiefe C.I., Canto J. (2009). Sex differences in mortality after acute myocardial infarction: Changes from 1994 to 2006. Arch. Intern. Med..

[4] Benamer H., Tafflet M., Bataille S., Escolano S., Livarek B., Fourchard V., Caussin C., Teiger E., Garot P., Lambert Y. (2011). Female gender is an independent predictor of in-hospital mortality after STEMI in the era of primary PCI: Insights from the greater Paris area PCI Registry. EuroIntervention.

[5] Stehli J., Martin C., Brennan A., Dinh D.T., Lefkovits J., Zaman S. (2019). Sex Differences Persist in Time to Presentation, Revascularization, and Mortality in Myocardial Infarction Treated With Percutaneous Coronary Intervention. J. Am. Heart Assoc..

[6] Lam C.S.P., Arnott C., Beale A.L., Chandramouli C., Hilfiker-Kleiner D., Kaye D.M., Ky B., Santema B.T., Sliwa K., Voors A.A. (2019). Sex differences in heart failure. Eur. Heart J..

[7] Kosmidou I., Redfors B., Selker H.P., Thiele H., Patel M.R., Udelson J.E., Magnus Ohman E., Eitel I., Granger C.B., Maehara A. (2017). Infarct size, left ventricular function, and prognosis in women compared to men after primary percutaneous coronary intervention in ST-segment elevation myocardial infarction: Results from an individual patient-level pooled analysis of 10 randomized trials. Eur. Heart J..

[8] Arora S., Stouffer G.A., Kucharska-Newton A.M., Qamar A., Vaduganathan M., Pandey A., Porterfield D., Blankstein R., Rosamond W.D., Bhatt D.L. (2019). Twenty Year Trends and Sex Differences in Young Adults Hospitalized With Acute Myocardial Infarction. Circulation.

[9] Lichtman J.H., Leifheit E.C., Safdar B., Bao H., Krumholz H.M., Lorenze N.P., Daneshvar M., Spertus J.A., D’Onofrio G. (2018). Sex Differences in the Presentation and Perception of Symptoms Among Young Patients with Myocardial Infarction: Evidence from the VIRGO Study (Variation in Recovery: Role of Gender on Outcomes of Young AMI Patients). Circulation.

[10] Bugiardini R., Manfrini O., Cenko E. (2019). Female sex as a biological variable: A review on younger patients with acute coronary syndrome. Trends Cardiovasc. Med..

[11] Cenko E., van der Schaar M., Yoon J., Kedev S., Valvukis M., Vasiljevic Z., Ašanin M., Miličić D., Manfrini O., Badimon L. (2019). Sex-Specific Treatment Effects After Primary Percutaneous Intervention: A Study on Coronary Blood Flow and Delay to Hospital Presentation. J. Am. Heart Assoc..

[12] Piro M., Della Bona R., Abbate A., Biasucci L.M., Crea F. (2010). Sex-related differences in myocardial remodeling. J. Am. Coll. Cardiol..

[13] Garcia M., Mulvagh S.L., Merz C.N., Buring J.E., Manson J.E. (2016). Cardiovascular Disease in Women: Clinical Perspectives. Circ. Res..

[14] Kessous R., Shoham-Vardi I., Pariente G., Holcberg G., Sheiner E. (2013). An association between preterm delivery and long-term maternal cardiovascular morbidity. Am. J. Obstet. Gynecol..

[15] Gianturco L., Bodini B.D., Atzeni F., Colombo C., Stella D., Sarzi-Puttini P., Drago L., Galaverna S., Turiel M. (2015). Cardiovascular and autoimmune diseases in females: The role of microvasculature and dysfunctional endothelium. Atherosclerosis.

[16] Mehta L.S., Watson K.E., Barac A., Beckie T.M., Bittner V., Cruz-Flores S., Dent S., Kondapalli L., Ky B., Okwuosa T. (2018). American Heart Association Cardiovascular Disease in Women and Special Populations Committee of the Council on Clinical Cardiology; Council on Cardiovascular and Stroke Nursing; and Council on Quality of Care and Outcomes Research. Cardiovascular Disease and Breast Cancer: Where These Entities Intersect: A Scientific Statement From the American Heart Association. Circulation.

[17] Wilson P.W., D’Agostino R.B., Sullivan L., Parise H., Kannel W.B. (2002). Overweight and obesity as determinants of cardiovascular risk: The Framingham experience. Arch. Intern. Med..

[18] Pizzi C., Rutjes A.W., Costa G.M., Fontana F., Mezzetti A., Manzoli L. (2011). Meta-analysis of selective serotonin reuptake inhibitors in patients with depression and coronary heart disease. Am. J. Cardiol..

[19] Raj S.R., Stein C.M., Saavedra P.J., Roden D.M. (2009). Cardiovascular effects of noncardiovascular drugs. Circulation.

[20] Cenko E., van der Schaar M., Yoon J., Manfrini O., Vasiljevic Z., Vavlukis M., Kedev S., Miličić D., Badimon L., Bugiardini R. (2019). Sex-Related Differences in Heart Failure After ST-Segment Elevation Myocardial Infarction. J. Am. Coll. Cardiol..

[21] Canali E., Masci P., Bogaert J., Bucciarelli Ducci C., Francone M., McAlindon E., Carbone I., Lombardi M., Desmet W., Janssens S. (2012). Impact of gender differences on myocardial salvage and post-ischaemic left ventricular remodelling after primary coronary angioplasty: New insights from cardiovascular magnetic resonance. Eur. Heart J. Cardiovasc. Imaging.

[22] D’Onofrio G., Safdar B., Lichtman J.H., Strait K.M., Dreyer R.P., Geda M., Spertus J.A., Krumholz H.M. (2015). Sex differences in reperfusion in young patients with ST-segment-elevation myocardial infarction: Results from the VIRGO study. Circulation.

[23] Chichareon P., Modolo R., Kerkmeijer L., Tomaniak M., Kogame N., Takahashi K., Chang C.C., Komiyama H., Moccetti T., Talwar S. (2020). Association of Sex With Outcomes in Patients Undergoing Percutaneous Coronary Intervention: A Subgroup Analysis of the GLOBAL LEADERS Randomized Clinical Trial. JAMA Cardiol..

[24] Kenkre T.S., Malhotra P., Johnson B.D., Handberg E.M., Thompson D.V., Marroquin O.C., Rogers W.J., Pepine C.J., Bairey Merz C.N., Kelsey S.F. (2017). Ten-Year Mortality in the WISE Study (Women’s Ischemia Syndrome Evaluation). Circ. Cardiovasc. Qual. Outcomes.

[25] Mega J.L., Hochman J.S., Scirica B.M., Murphy S.A., Sloan S., McCabe C.H., Merlini P., Morrow D.A. (2010). Clinical features and outcomes of women with unstable ischemic heart disease: Observations from metabolic efficiency with ranolazine for less ischemia in non-ST-elevation acute coronary syndromes-thrombolysis in myocardial infarction 36 (MERLIN-TIMI 36). Circulation.

[26] Otten A.M., Maas A.H., Ottervanger J.P., Kloosterman A., van‘t Hof A.W., Dambrink J.H., Gosselink A.T., Hoorntje J.C., Suryapranata H., de Boer M.J. (2013). Is the difference in outcome between men and women treated by primary percutaneous coronary intervention age dependent? Gender difference in STEMI stratified on age. Eur. Heart J. Acute Cardiovasc. Care..

[27] Lawless M., Appelman Y., Beltrame J.F., Navarese E.P., Ratcovich H., Wilkinson C., Kunadian V. (2023). Sex differences in treatment and outcomes amongst myocardial infarction patients presenting with and without obstructive coronary arteries: A prospective multicentre study. Eur. Heart J. Open.

[28] Canton L., Fedele D., Bergamaschi L., Foà A., Di Iuorio O., Tattilo F.P., Rinaldi A., Angeli F., Armillotta M., Sansonetti A. (2023). Sex- and age-related differences in outcomes of patients with acute myocardial infarction: MINOCA vs. MIOCA. Eur. Heart J. Acute Cardiovasc. Care.

[29] Lindahl B., Baron T., Albertucci M., Prati F. (2021). Myocardial infarction with non-obstructive coronary artery disease. EuroIntervention.

[30] Wei J., Cheng S., Bairey Merz C.N. (2019). Coronary Microvascular Dysfunction Causing Cardiac Ischemia in Women. JAMA.

[31] Tamis-Holland J.E., Jneid H., Reynolds H.R., Agewall S., Brilakis E.S., Brown T.M., Lerman A., Cushman M., Kumbhani D.J., Arslanian-Engoren C. (2019). Contemporary Diagnosis and Management of Patients With Myocardial Infarction in the Absence of Obstructive Coronary Artery Disease: A Scientific Statement From the American Heart Association. Circulation.

[32] Mehta P.K., Huang J., Levit R.D., Malas W., Waheed N., Bairey Merz C.N. (2022). Ischemia and no obstructive coronary arteries (INOCA): A narrative review. Atherosclerosis.

[33] Pelliccia F., Camici P.G. (2023). Updates on MINOCA and INOCA through the 2022 publications in the International Journal of Cardiology. Int. J. Cardiol..

[34] Ferry A.V., Anand A., Strachan F.E., Mooney L., Stewart S.D., Marshall L., Chapman A.R., Lee K.K., Jones S., Orme K. (2019). Presenting Symptoms in Men and Women Diagnosed With Myocardial Infarction Using Sex-Specific Criteria. J. Am. Heart Assoc..

[35] Angeli F., Ricci F., Moscucci F., Sciomer S., Bucciarelli V., Bianco F., Mattioli A.V., Pizzi C., Gallina S. (2024). Sex- and gender-related disparities in chest pain syndromes: The feminine mystique of chest pain. Curr. Probl. Cardiol..

[36] Taqueti V.R., Solomon S.D., Shah A.M., Desai A.S., Groarke J.D., Osborne M.T., Hainer J., Bibbo C.F., Dorbala S., Blankstein R. (2018). Coronary microvascular dysfunction and future risk of heart failure with preserved ejection fraction. Eur. Heart J..

[37] Mallat Z., Fornes P., Costagliola R., Esposito B., Belmin J., Lecomte D., Tedgui A. (2001). Age and gender effects on cardiomyocyte apoptosis in the normal human heart. J. Gerontol. A Biol. Sci. Med. Sci..

[38] Biondi-Zoccai G.G., Abate A., Bussani R., Camilot D., Giorgio F.D., Marino M.P., Silvestri F., Baldi F., Biasucci L.M., Baldi A. (2005). Reduced post-infarction myocardial apoptosis in women: A clue to their different clinical course?. Heart.

[39] Sofia R.R., Serra A.J., Silva JAJr Antonio E.L., Manchini M.T., Oliveira F.A., Teixeira V.P., Tucci P.J. (2014). Gender-based differences in cardiac remodeling and ILK expression after myocardial infarction. Arq. Bras. Cardiol..

[40] Guerra S., Leri A., Wang X., Finato N., Di Loreto C., Beltrami C.A., Kajstura J., Anversa P. (1999). Myocyte death in the failing human heart is gender dependent. Circ. Res..

[41] Iorga A., Cunningham C.M., Moazeni S., Ruffenach G., Umar S., Eghbali M. (2017). The protective role of estrogen and estrogen receptors in cardiovascular disease and the controversial use of estrogen therapy. Biol. Sex. Differ..

[42] Duca F., Zotter-Tufaro C., Kammerlander A.A., Aschauer S., Binder C., Mascherbauer J., Bonderman D. (2018). Gender-related differences in heart failure with preserved ejection fraction. Sci. Rep..

[43] Kessler E.L., Rivaud M.R., Vos M.A., van Veen T.A.B. (2019). Sex-specific influence on cardiac structural remodeling and therapy in cardiovascular disease. Biol. Sex. Differ..

[44] McMurray J.J.V., Jackson A.M., Lam C.S.P., Redfield M.M., Anand I.S., Ge J., Lefkowitz M.P., Maggioni A.P., Martinez F., Packer M. (2020). Effects of Sacubitril-Valsartan Versus Valsartan in Women Compared With Men With Heart Failure and Preserved Ejection Fraction: Insights From PARAGON-HF. Circulation.

[45] Mann D.L., Bogaev R., Buckberg G.D. (2010). Cardiac remodelling and myocardial recovery: Lost in translation?. Eur. J. Heart Fail..

[46] French B.A., Kramer C.M. (2007). Mechanisms of Post-Infarct Left Ventricular Remodeling. Drug Discov. Today Dis. Mech..

[47] Conte LFabiani I., Barleta V., Giannini C., Leo L.A., Delle Done M.G., Palagi C., Nardi C., Dini F.L., Petronio A.S., Marzilli M. (2012). The role of cardiovascular imaging to understand the different patterns of post-ischemic remodeling. J. Cardiovasc. Echocardiogr..

[48] Miller R.J.H., Mikami Y., Heydari B., Wilton S.B., James M.T., Howarth A.G., White J.A., Lydell C.P. (2020). Sex-specific relationships between patterns of ventricular remodelling and clinical outcomes. Eur. Heart J. Cardiovasc. Imaging.

[49] Verma A., Meris A., Skali H., Ghali J.K., Arnold J.M., Bourgoun M., Velazquez E.J., McMurray J.J., Kober L., Pfeffer M.A. (2008). Prognostic implications of left ventricular mass and geometry following myocardial infarction: The VALIANT (VALsartan In Acute myocardial iNfarcTion) Echocardiographic Study. JACC Cardiovasc. Imaging.

[50] Lam C.S., McEntegart M., Claggett B., Liu J., Skali H., Lewis E., Køber L., Rouleau J., Velazquez E., Califf R. (2015). Sex differences in clinical characteristics and outcomes after myocardial infarction: Insights from the Valsartan in Acute Myocardial Infarction Trial (VALIANT). Eur. J. Heart Fail..

[51] Cunningham J.W., Vaduganathan M., Claggett B.L., John J.E., Desai A.S., Lewis E.F., Zile M.R., Carson P., Jhund P.S., Kober L. (2020). Myocardial Infarction in Heart Failure With Preserved Ejection Fraction: Pooled Analysis of 3 Clinical Trials. JACC Heart Fail..

[52] Kim G.H., Uriel N., Burkhoff D. (2018). Reverse remodelling and myocardial recovery in heart failure. Nat. Rev. Cardiol..

[53] Rodrigues P.G., Leite-Moreira A.F., Falcão-Pires I. (2016). Myocardial reverse remodeling: How far can we rewind?. Am. J. Physiol. Heart Circ. Physiol..

[54] Arata A., Ricci F., Khanji M.Y., Mantini C., Angeli F., Aquilani R., Di Baldassarre A., Renda G., Mattioli A.V., Nodari S. (2023). Sex Differences in Heart Failure: What Do We Know?. J. Cardiovasc. Dev. Dis..

[55] Countouris M.E., Villanueva F.S., Berlacher K.L., Cavalcante J.L., Parks W.T., Catov J.M. (2021). Association of Hypertensive Disorders of Pregnancy With Left Ventricular Remodeling Later in Life. J. Am. Coll. Cardiol..

[56] Ricci F., De Innocentiis C., Verrengia E., Ceriello L., Mantini C., Pietrangelo C., Irsuti F., Gabriele S., D’Alleva A., Khanji M.Y. (2020). The Role of Multimodality Cardiovascular Imaging in Peripartum Cardiomyopathy. Front. Cardiovasc. Med..

[57] Geske J.B., Ong K.C., Siontis K.C., Hebl V.B., Ackerman M.J., Hodge D.O., Miller V.M., Nishimura R.A., Oh J.K., Schaff H.V. (2017). Women with hypertrophic cardiomyopathy have worse survival. Eur. Heart J..

[58] Eng C.M., Fletcher J., Wilcox W.R., Waldek S., Scott C.R., Sillence D.O., Breunig F., Charrow J., Germain D.P., Nicholls K. (2007). Fabry disease: Baseline medical characteristics of a cohort of 1765 males and females in the Fabry Registry. J. Inherit. Metab. Dis..

[59] Zampieri M., Argirò A., Allinovi M., Tassetti L., Zocchi C., Gabriele M., Andrei V., Fumagalli C., Di Mario C., Tomberli A. (2022). Sex-related differences in clinical presentation and all-cause mortality in patients with cardiac transthyretin amyloidosis and light chain amyloidosis. Int. J. Cardiol..

[60] Ku L., Feiger J., Taylor M., Mestroni L., Familial Cardiomyopathy Registry (2003). Cardiology patient page. Familial dilated cardiomyopathy. Circulation.

[61] Hollan I., Meroni P.L., Ahearn J.M., Cohen Tervaert J.W., Curran S., Goodyear C.S., Hestad K.A., Kahaleh B., Riggio M., Shields K. (2013). Cardiovascular disease in autoimmune rheumatic diseases. Autoimmun. Rev..

[62] Santema B.T., Ouwerkerk W., Tromp J., Sama I.E., Ravera A., Regitz-Zagrosek V., Hillege H., Samani N.J., Zannad F., Dickstein K. (2019). Identifying optimal doses of heart failure medications in men compared with women: A prospective, observational, cohort study. Lancet.

[63] Fairweather D., Beetler D.J., Musigk N., Heidecker B., Lyle M.A., Cooper L.T., Bruno K.A. (2023). Sex and gender differences in myocarditis and dilated cardiomyopathy: An update. Front. Cardiovasc. Med..

[64] Fenech A.G., Magri V.P. (2020). Gender Differences in Drug Therapy. Drug Discovery and Evaluation: Methods in Clinical Pharmacology.

[65] Hsich E.M. (2019). Sex Differences in Advanced Heart Failure Therapies. Circulation.

[66] Eifert S., Kofler S., Nickel T., Horster S., Bigdeli A.K., Beiras-Fernandez A., Meiser B., Kaczmarek I. (2012). Gender-based analysis of outcome after heart transplantation. Exp. Clin. Transplant..

[67] Linde C., Cleland J.G.F., Gold M.R., Claude Daubert J., Tang A.S.L., Young J.B., Sherfesee L., Abraham W.T. (2018). The interaction of sex, height, and QRS duration on the effects of cardiac resynchronization therapy on morbidity and mortality: An individual-patient data meta-analysis. Eur. J. Heart Fail..

[68] Medzikovic L., Azem T., Sun W., Rejali P., Esdin L., Rahman S., Dehghanitafti A., Aryan L., Eghbali M. (2023). Sex Differences in Therapies against Myocardial Ischemia-Reperfusion Injury: From Basic Science to Clinical Perspectives. Cells.

[69] Tamargo J., Rosano G., Walther T., Duarte J., Niessner A., Kaski J.C., Ceconi C., Drexel H., Kjeldsen K., Savarese G. (2017). Gender differences in the effects of cardiovascular drugs. Eur. Heart J. Cardiovasc. Pharmacother..

[70] Ali Z., Landmesser U., Karimi Galougahi K., Maehara A., Matsumura M., Shlofmitz R.A., Guagliumi G., Price M.J., Hill J.M., Akasaka T. (2021). Optical coherence tomography-guided coronary stent implantation compared to angiography: A multicentre randomised trial in PCI—Design and rationale of ILUMIEN IV: OPTIMAL PCI. EuroIntervention.

[71] Gerdts E., Regitz-Zagrosek V. (2019). Sex differences in cardiometabolic disorders. Nat. Med..

[72] Navarro S., Talucci I., Göb V., Hartmann S., Beck S., Orth V., Stoll G., Maric H.M., Stegner D., Nieswandt B. (2024). The humanized platelet glycoprotein VI Fab inhibitor EMA601 protects from arterial thrombosis and ischaemic stroke in mice. Eur. Heart J..

[73] Zuriaga M.A., Yu Z., Matesanz N., Truong B., Ramos-Neble B.L., Asensio-López M.C., Uddin M.M., Nakao T., Niroula A., Zorita V. (2024). Colchicine prevents accelerated atherosclerosis in TET2-mutant clonal haematopoiesis. Eur. Heart J..

[74] Titova O.E., Yuan S., Byberg L., Baron J.A., Lind L., Michaëlsson K., Larsson S.C. (2024). Plasma proteome and incident myocardial infarction: Sex-specific differences. Eur. Heart J..

[75] Crea F. (2024). Ischaemic heart disease: Focus on sex-related differences and novel therapeutic targets. Eur. Heart J..

[76] Kresoja K.P., Rubini Giménez M., Thiele H. (2024). The SEX-SHOCK score-the emperor’s new clothes?. Eur. Heart J..

[77] Wang Y., Zeller M., Auffret V., Georgiopoulos G., Räber L., Roffi M., Templin C., Muller O., Liberale L., Ministrini S. (2024). Sex-specific prediction of cardiogenic shock after acute coronary syndromes: The SEX-SHOCK score. Eur. Heart J..

[78] Klotz S., Danser A.H., Foronjy R.F., Oz M.C., Wang J., Mancini D., D’Armiento J., Burkhoff D. (2007). The impact of angiotensin-converting enzyme inhibitor therapy on the extracellular collagen matrix during left ventricular assist device support in patients with end-stage heart failure. J. Am. Coll. Cardiol..

[79] Franconi F., Campesi I. (2014). Pharmacogenomics, pharmacokinetics and pharmacodynamics: Interaction with biological differences between men and women. Br. J. Pharmacol..

[80] Rathore S.S., Wang Y., Krumholz H.M. (2002). Sex-based differences in the effect of digoxin for the treatment of heart failure. N. Engl. J. Med..

[81] Solomon S.D., Claggett B., Lewis E.F., Desai A., Anand I., Sweitzer N.K., O’Meara E., Shah S.J., McKinlay S., Fleg J.L. (2016). Influence of ejection fraction on outcomes and efficacy of spironolactone in patients with heart failure with preserved ejection fraction. Eur. Heart J..

